# The Making of a Modern Hospital

**Published:** 1911-10-07

**Authors:** 


### THE MAKING OF A MODERN HOSPITAL.

### I.—Introductory.

The present time is one of unrest in the hospital world. There is talk of reform and alteration; even the system that has served us so long and so efficiently has come in for criticism which condemns before it rightly comprehends. The public, as a whole, and even hospital workers who are presumably acquainted with the ins and outs of the question, reveal, by their expressions of opinion, the fact that they are not fully aware of the intricacies of the great problem of hospital management, construction, and administration. There is no stagnation in the hospital world. Everything tends to progress, and the evaluation of new systems, new models, new methods, goes on from day to day. With this progressive advance few workers can, unaided and by themselves, deal adequately. The literature is so scattered, so voluminous, and so technical for the most part, that interpretations and signposts are constantly demanded. Those of us who take a knowledgeable, active, and working interest in hospital problems are always eager to hear of, and examine, suggested improvements which will tend to better our existing institutions. No one of us is so advanced and so encyclopædic in his knowledge of hospital matters that he has nothing to learn from others. The difficulty is to collate the information, and to present a broad survey of the field that will be an indication of the extent of the progress that has been achieved so far, and the outlines of the amendments which have been proposed for the future.

It is our intention in The Hospital to give our readers such a detailed sketch of modern advances in hospital work. With this intention in view we commissioned, more than four years ago, a member of our staff who is fully acquainted with hospital methods in this country to undertake an extensive investigation into the systems that prevail elsewhere, and to report on them for the benefit of our readers. In former issues of this journal preliminary reports from our Special Commissioner have from time to time been published. These dealt with isolated examples of modern hospital construction and management, and, more recently, with the special features of the German system, especially in so far as it concerned the relation between the hospital and social legislation. We propose to continue this series of articles, but in addition to publish, under the title that heads this introductory article, a number of reports dealing with the general advances in hospital work that have been made during the last ten years. Such a *résumé* will, we think, be of interest to our readers, not only to those who are actively engaged in hospital work but equally to those who, as committeemen or as friends of their own institutions, watch the progress and development of our hospitals with that appreciative concern which is generated by the conviction that our system has been the means of benefiting the community to an incalculable extent.

As a preface to the series it is permissible to outline briefly the general scope and character of the articles which we hope to publish from time to time. Our Commissioner has personally inspected and gone fully into the details of administration and management of many of the newest and most up-to-date hospitals on the Continent and in America, and has compared the results obtained in this manner with those available in the published reports of colonial institutions. He has taken care to get first-hand information on all points and has satisfied himself that such information was reliable and trustworthy by hearing the views of different authorities who were competent to express an opinion. His, and our, thanks are due to the managers, superintendents, secretaries, medical staffs, and lay helpers who have aided him in this task, and as it is obviously impossible to thank them all individually we tender hereby to them collectively our appreciation of their assistance. The willingness to give information, and the readiness to allow a stranger colleague to inspect their institutions, are surely the best proofs of the fact that they were eager to place at the disposal of fellow workers in this country the results of the experience they had gained themselves. For that assistance we are most grateful.

The plan on which these articles will be based is simple, for a summary such as we propose giving must be elemental, and elementals must be handled with directness and simplicity in order to avoid confusion. It is our intention, then, to take as a type of the modern hospital a combination of departments and to show, by illustrations from the best constructions yet erected, how this model type may be composed. It is only after due consideration and thought that we have decided to adopt this plan from the many alternative schemes of treatment that suggested themselves. It would have been comparatively easy to take one or two principal institutions as models and to show in what manner they fulfil, or fail to fulfil, the requirements of the ideal hospital. But this selective method, we venture to think, would not have been of such comparative value to our readers who are interested in the subject as the one we have finally chosen. We will take, as an example, the question of the ideal site—a matter of considerable, one may even go so far as to say of primary, importance in hospital construction. By describing the difficulties which have been experienced in one or two typical cases, and showing how these have been overcome, and further by comparing the different sites of certain model institutions, we enable the reader to obtain a succinct epitome of the question of the consideration of sites. By showing, again, the factors which have tended in different institutions all over the world to raise the daily expenditure per bed, we similarly put the hospital worker in a position, with these data in front of him, to realise the progressive costliness of modern hospitals. By dealing in the same manner with special departments, we offer a criterion which may be applied by every institutional manager to his own hospital, and give a summary of the development in lines of treatment which modern medicine with all its refinements has brought about. It is only by comparison and contrast that such a summary can be made of real and practical value, and that means of contrast and comparison we propose to give in the series of articles to follow.

Needless to say, such an epitome of hospital construction and progress cannot be absolutely complete. There is no finality in a subject that is unstable by reason of the daily changes that occur in methods of creation and adaptation. Nor must it be understood that the statements advanced in this series are dogmatic. So far as is possible, our Commissioner will confine himself to questions of fact—to data only. Expressions of opinion which must necessarily crop up from time to time will only be made on data from which the reader may draw his own conclusions. We would impress on hospital workers the fact that discussion of such data is always illuminative and instructive, and we would therefore welcome such discussion in the reasonable hope that it may lead to tangible results. It is impossible, in an investigation which has extended over several years, to bear in mind the alterations and improvements which have been made from time to time in individual institutions. Where such improvements have been made and have escaped the view of our Commissioner, we trust hospital workers will supply the hiatus and communicate with us, drawing our attention to the fact. Only in that manner can the series be made really up-to-date and practically effective as a summary of modern hospital work.

One other point we would lay stress on, and it is this: The interests of the hospital and those of the community that supply what is technically known as its "population" are identical. This is a fact often overlooked. There is a tendency, fostered by indiscriminate cavilling at the faults and deficiencies of particular institutions, to inveigle the public into the belief that these interests are antagonistic, that the hospital blocks instead of paves the way to social improvement. To hospital workers we need not demonstrate the fallacy of such reasoning, but it is obvious that it needs refutation to the public ignorant of the difficulties of modern hospital work. The present series of articles is an attempt to set in a broad light the salient points of that work before the public; not merely the hospital public within the walls of our institutions, but the large outside public from which our hospitals draw support, praise, or blame. We therefore appeal to hospital workers to interest themselves in making the facts contained in these articles known to those who are subscribers to their institutions. By creating a healthy interest in hospital work—an interest that acknowledges, from knowledge of the facts, the progressive difficulties with which the modern hospital has to cope, and that makes due allowance for such difficulties in construction, administration, treatment, and upkeep—the hospitals will inevitably gain in appreciation, in credit, in service, and in finance. We feel sure that a large section of the public only awaits the development of that stimulative interest to respond more wholeheartedly to the appeal for help and personal service which emanates from our voluntary institutions. That being the case, we offer these summaries to our readers in the hope that they will occasion a concentration of energy upon the discussion of the problems that at present confront hospital workers in this country. The time is opportune for such a discussion; what is wanting are the data on which the conclusions ultimately to be arrived at must be based. We cannot flatter ourselves that we shall be able to supply all those data, but we can at least endeavour to give so much that the deductive process will be materially simplified.

